# Characterization of nitrate use efficiency in tea plant (*Camellia sinensis*) based on leaf chlorate sensitivity

**DOI:** 10.1093/hr/uhae354

**Published:** 2024-12-28

**Authors:** Wenjing Zhang, Xiaoying Dong, Kang Ni, Lifeng Ma, Lizhi Long, Jianyun Ruan

**Affiliations:** Key Laboratory of Biology, Genetics and breeding of Special Economic Animals and Plants, Ministry of Agriculture and Rural Affairs, 310008, No.9 Meiling South Road, Hangzhou, Xihu District, Zhejiang ProvinceP.R.China; Graduate School of Chinese Academy of Agricultural Sciences, 100081, No.12 Zhongguancun South Street, Haidian District, BeijingP.R.China; Key Laboratory of Biology, Genetics and breeding of Special Economic Animals and Plants, Ministry of Agriculture and Rural Affairs, 310008, No.9 Meiling South Road, Hangzhou, Xihu District, Zhejiang ProvinceP.R.China; Graduate School of Chinese Academy of Agricultural Sciences, 100081, No.12 Zhongguancun South Street, Haidian District, BeijingP.R.China; Key Laboratory of Biology, Genetics and breeding of Special Economic Animals and Plants, Ministry of Agriculture and Rural Affairs, 310008, No.9 Meiling South Road, Hangzhou, Xihu District, Zhejiang ProvinceP.R.China; Xihu National Agricultural Experimental Station for Soil Quality, Hangzhou 310008, China; Key Laboratory of Biology, Genetics and breeding of Special Economic Animals and Plants, Ministry of Agriculture and Rural Affairs, 310008, No.9 Meiling South Road, Hangzhou, Xihu District, Zhejiang ProvinceP.R.China; Xihu National Agricultural Experimental Station for Soil Quality, Hangzhou 310008, China; Key Laboratory of Biology, Genetics and breeding of Special Economic Animals and Plants, Ministry of Agriculture and Rural Affairs, 310008, No.9 Meiling South Road, Hangzhou, Xihu District, Zhejiang ProvinceP.R.China; Xihu National Agricultural Experimental Station for Soil Quality, Hangzhou 310008, China; Key Laboratory of Biology, Genetics and breeding of Special Economic Animals and Plants, Ministry of Agriculture and Rural Affairs, 310008, No.9 Meiling South Road, Hangzhou, Xihu District, Zhejiang ProvinceP.R.China; Xihu National Agricultural Experimental Station for Soil Quality, Hangzhou 310008, China

## Abstract

Nitrate (NO_3_^−^), a key form of inorganic nitrogen (N) in soils, is typically lost in tea gardens through leaching. However, NO_3_^−^ utilization efficiency (NiUE) and its characteristic mechanism in tea plants remain unclear. This study screened contrastive genotypes of NiUE using leaf chlorate sensitivity and explored the potential genes that regulate this process. Fresh branches of 10 cultivars were hydroponically cultivated and subjected to potassium nitrate (KNO_3_) and potassium chlorate (KClO_3_) treatments, with the former as the control group. The sensitive cultivar, Zhenong 117 (ZN117), showed a decrease in SPAD and F_v_/F_m_ values following KClO_3_ treatment, while the tolerant cultivar, Teiguanyin (TGY), exhibited minimal significant changes. After 5 days of cultivation, the ^15^N concentration and proportion in new shoots of ZN117 were significantly higher than those in TGY. Transcriptome analysis revealed that the expression of genes responsible for NO_3_^−^ transport, including the nitrate transporters *NRT2.4*, *NPF4.6*, *NPF6.1*, *NPF1.10*, and *NPF1.11*, significantly increased in ZN117 after NO_3_^−^ supply. Genes involved in NO_3_^−^ reduction, chlorophyll synthesis, and photosynthesis were progressively induced. Coexpression network analysis indicated that the squamosa promoter-binding protein activated the onset of NO_3_^−^ signaling, while basic helix–loop–helix transcripts were triggered to higher levels during NO_3_^−^ supply. This study proposes a rapid characterization method of NiUE in woody plants and a speculative molecular regulatory mechanism for the NO_3_^−^ transfer and remobilization of tea plants. A set of specific genes involved in NO_3_^−^ transport, reduction, and mobilization were identified and proposed as marker genes for NiUE in tea plants.

## Introduction

Nitrogen (N) is the most abundant element in the Earth’s atmosphere and is essential for plant growth. Plants acquire substances containing N, such as nitrate (NO_3_^−^), ammonium (NH_4_^+^), and amino acids (AAs) from the soil to support their basic life processes throughout all stages of development [1]. The tea plant (*Camellia sinensis*) is an evergreen woody crop primarily cultivated for its leaves, which are rich in various bioactive components [2]. Theanine is responsible for the umami taste in tea. Its content, an important criterion for the evaluation of tea quality, is largely affected by the N status of plants [3]. However, the limited available N sources in soils leads to farmers commonly applying high doses of N fertilizers to achieve a high accumulation of AAs in tea leaves. The application rate of N fertilizer for tea plants is 5–6 times higher than the global average rate for grain crops, and NO_3_^−^ is one of the main sources of N, after NH_4_^+^, absorbed by tea plants [4]. Moreover, tea plantations are often located in regions with high rainfall, where NO_3_^−^ from excessive N fertilizer application is prone to leaching and denitrification, resulting in water eutrophication and the emission of nitrous oxide [5, 6]. Thus, increasing nitrate utilization efficiency (NiUE) is crucial for reducing the usage of N fertilizers, thereby establishing a sustainable tea production system.

Previous studies on tea plants have identified methods to evaluate NiUE-related traits based on biomass accumulation, ion influx kinetics, the contents of relevant biochemical components, and gene expression profiles [7–10]. However, thesemethods are time-consuming and are easily influenced by environmental changes. NO_3_^−^ absorption and transportation in higher plants are mainly facilitated by nitrate transporters (NRTs) [11]. NO_3_^−^ is first reduced into nitrite (NO_2_^−^) in the cytoplasm, then further reduced into NH_4_^+^ in the plastid, and finally assimilated into AAs in either plastid or cytoplasm [12]. Generally, plants cannot distinguish between chlorate ions (ClO_3_^−^) and NO_3_^−^ during transport and reduction processes due to their similar chemical structure. ClO_3_^−^ enters cells via the same transporters as NO_3_^−^ and is subsequently reduced to chlorite (ClO_2_^−^) by nitrate reductase (NR) [13]. ClO_2_^−^ is toxic to plants and induces chlorotic discolorations [14]. Therefore, ClO_3_^−^ serves as an analogic tracer for NO_3_^−^ and provides a sensitive and rapid method for the screening of NiUE in plants. The ClO_3_^−^-sensitive assay has been successfully applied to cereal crops such as barley (*Hordeum vulgare*) [15], rice (*Oryza sativa*) [16, 17], and wheat (*Triticum aestivum*) [18]. However, whether the ClO_3_^−^-resistance assay can be used to assess the NO_3_^−^ utilization rate in woody plants for rapid screening, particularly using branches instead of whole plants, is unclear.

Understanding the regulation of N uptake and assimilation in tea plants is crucial for improving nitrogen use efficiency (NUE), and can thus aid in reducing nitrogen fertilizer application in tea production. Several genes involved in N transport and metabolism have been identified in tea plants, including *CsAMT1.2*, *CsNRT2.4*, *CsAAP1*, *CsGS1.1*, *CsTSI*, and *CsGDH2.1*. These genes play important roles in N absorption and AA biosynthesis, evidenced by the combination of omics data and molecular biotechnologies [10, 19–23]. However, the regulation and coordination of these genes with the processes of NO_3_^−^ reduction and assimilation are still not fully understood in tea plants. Recent studies in other plant species have identified several transcription factors (TFs) involved in the dynamic activation of NO_3_^−^ signal transduction. For example, in *Arabidopsis*, *AtNLP7* acts as a master positive regulator, activated by NO_3_^−^ signal from the cytoplasm to the nuclear, thereby triggering instant responses to N availability [24]. In contrast, *AtLBD37/38/39*, as a negative regulator, can repress genes involved in NO_3_^−^ transport and reduction [25]. In rice, the degradation of SPX4 promotes the translocation of *OsNLP3*, a homolog of *AtNLP7*, thus transmitting the NO_3_^−^ signal to the nucleus [26]. In addition, *OsMADS25* has been found to interact with *OsNAR2.1* in response to NO_3_^−^ signaling, thereby moving from the cytoplasm to the nucleus and activating the expressions of *OsMADS27*, *OsARF7*, and *OsNRT2.1/2.2/2.3a* to positively regulate root development in the presence of NO_3_^−^ [27,28]. However, the specific TFs and their regulatory roles in NO_3_^−^ signaling in tea plants are still unknown.

This study explores a high-throughput method for NiUE screening in woody perennial plants based on ClO_3_^−^ resistance and attempts to determine the molecular regulatory network of NO_3_^−^ transport and utilization in varieties with high NiUE. Ten representative tea plant cultivars from various tea regions in China with unique leaf colors and considered suitable for processing into different types of tea beverages were selected in this experiment. The resistance to ClO_3_^−^ was measured by daily changes in SPAD and F_v_/F_m_ values. The SPAD value corresponds to the amount of chlorophyll in the sample leaf [29]. F_v_/F_m_ is a robust indicator of the maximum quantum yield of photosystem II complex (PSII). Any type of stress results in inactivated damage to PSII and reduces the F_v_/F_m_ value [30]. Therefore, changes in these two values can reflect the speed and degree of leaf injury after ClO_3_^−^ treatment. The more sensitive the leaves are to ClO_3_^−^, the higher the NiUE of this variety. Our results reveal that the chlorate-sensitive cultivar, ZN117, exhibited a higher capacity for NO_3_^−^ transport and utilization compared to the chlorate-tolerant cultivar, TGY. Furthermore, by analyzing the time-course transcriptome profiles of leaves in two contrastive cultivars under KNO_3_ and KClO_3_ treatments, we proposed a speculative molecular framework for NO_3_^−^ remobilization. Based on these results, we identified the genes involved in *CsNR* (nitrate reductase), *CsNiR* (nitrite reductase), and *CsNRTs* (nitrate transporters), including *CsNPF1.10*, *CsNPF1.11*, *CsNPF4.6*, *CsNPF6.1*, and *CsNRT2.4*. These genes may potentially play major roles in NO_3_^−^ transport and utilization in tea plants. Furthermore, *bHLH*, *Fd-NiR*, and *NPF2.2* may serve as marker genes to detect NiUE as determined in a wider range of genotypes. Overall, our study provides valuable insights into the molecular regulatory network of tea plants with a high NiUE genotype. Further research is required to validate the identified marker genes and fully understand their roles in NO_3_^−^ signaling and remobilization in tea plants, which is of great significance for breeding and genetic improvement [31].

## Results

### Chlorate sensitivity of 10 cultivars

**Figure 1:**
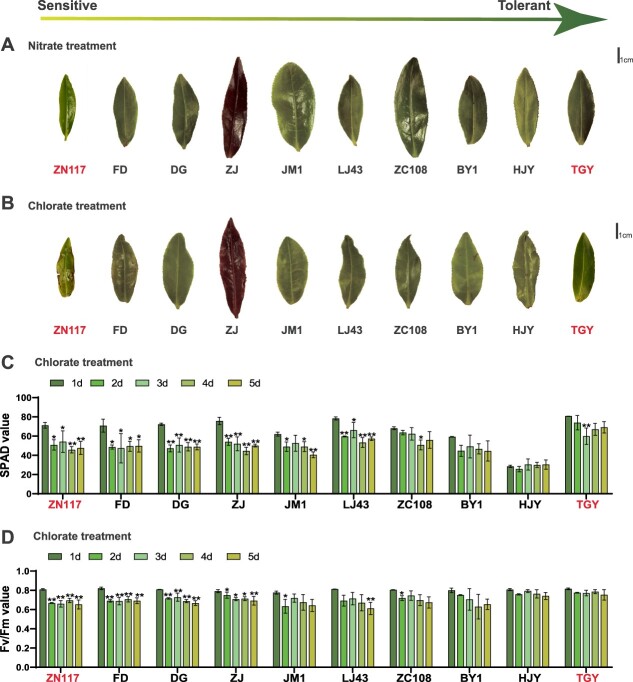
Leaf phenotype and physiological changes after nitrate and chlorate treatments of 10 cultivars. Leaf phenotype after 5-day (A) nitrate and (B) chlorate treatments. Temporal changes in (C) SPAD and (D) F_v_/F_m_ values after chlorate treatment. Bars represent the mean ± standard deviation (SD) (*n* = 3) with *t*-tests at ^*^P < 0.05 and ^**^P < 0.01. BY1, Baiye 1; DG, Dangui; FD, Fuding Dabaicha; HJY, Huangjinya; LJ43, Longjing 43; TGY, Tieguanyin; JM1, Jiaming 1; ZC108, Zhongcha 108; ZJ, Zijuan; and ZN117, Zhenong 117.

The third leaf of branches from 10 cultivars exhibited varying phenotypic changes after exposure to toxic KClO_3_. The leaves of ZN117 displayed mottled yellowing and withering at the edges, while in other cultivars, the changes in the leaves were less pronounced (Fig. 1a, b). The SPAD and F_v_/F_m_ values of ZN117 decreased faster after the chlorate treatment compared to the nitrate treatment. For TGY, the daily declining frequency of SPAD and F_v_/F_m_ under both treatments was essentially similar in branch hydroponic culture conditions. The SPAD and F_v_/F_m_ values of ZN117 began to decline significantly after 2 days of chlorate treatment. In contrast, there was no significant reduction in F_v_/F_m_ in the nitrate group. Moreover, the F_v_/F_m_ value did not decline significantly in TGY under the chlorate and nitrate treatments (Fig. 1c, d and Fig. S1, Tables S1, 2).

**Figure 2:**
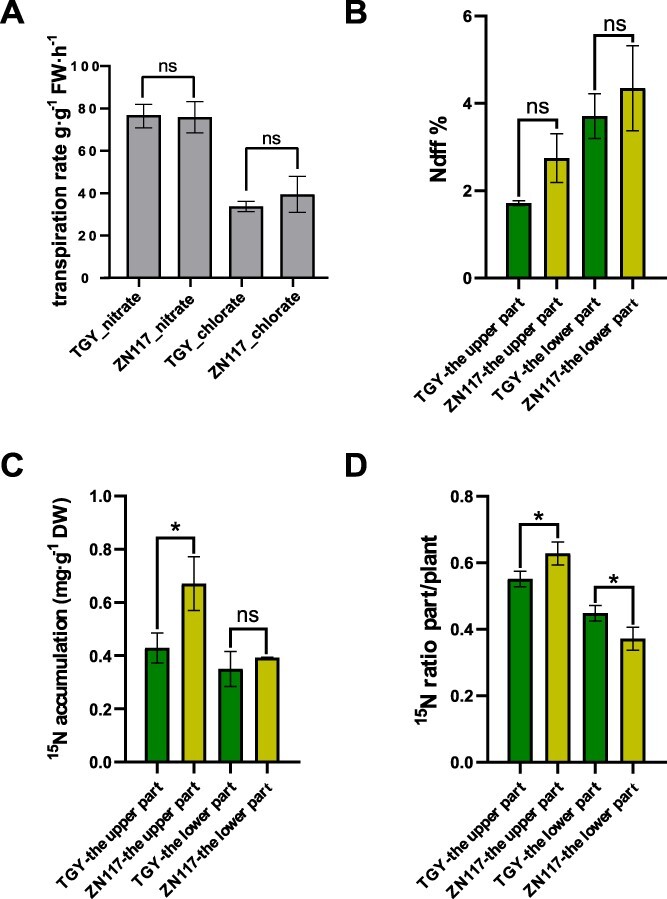
Transpiration rates and K^15^NO3 isotopic tracing results in ZN117 and TGY. (A) Transpiration rates observed in both cultivars after treatments; (B) N_dff_%, (C) ^15^N contents, and (D) ^15^N ratio changes in the upper and lower parts in both cultivars after treatments. Bars represent the mean ± standard error (SE) (*n* = 3) with *t*-tests at ^*^P < 0.05 and ^**^P < 0.01.

ZN117 and TGY were selected for further analysis based on the changes in the SPAD and F_v_/F_m_ values. The transpiration rate of the chlorate group was lower than that of the nitrate group, but the difference was not significant (Fig. 2a). In addition, there was no notable variation in N_dff_% between TGY and ZN117 (Fig. 2b). The ^15^N accumulation in new shoots of ZN117 was markedly greater than that of TGY, while no difference was observed in the lignified stem section (Fig. 2c). The ^15^N ratio of the new shoots and stem of ZN117 was significantly higher and lower than those of TGY, respectively, suggesting that ZN117 had a greater ^15^N uptake and mobilization to new shoots compared to TGY (Fig. 2d).

### Transcriptome analyses of TGY and ZN117 after nitrate and chlorate treatments

**Figure 3:**
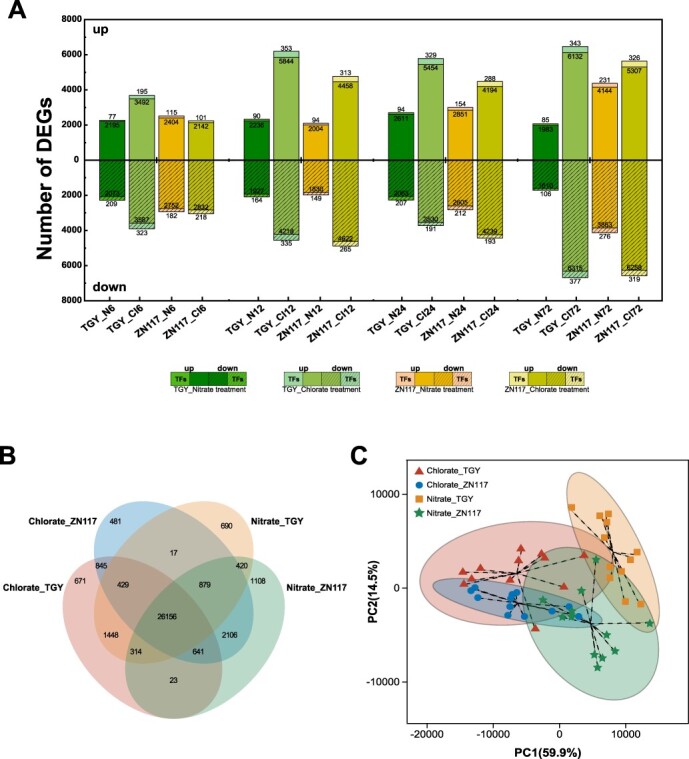
Overview of transcriptional profiles. (A) Number of upregulated (upper bars) and downregulated (lower bars) DEGs in TGY and ZN117 under the nitrate (N) and (B) chlorate (Cl) treatments. (B) Number of overlapping DEGs of the treatment groups. (C) PCA analysis of DEGs of the treatment groups. DEGs were determined by FDR ≤ 0.01 and fold change >1.5 using DESeq2.

The leaves of ZN117 and TGY were collected at five time points and subjected to nitrate and chlorate treatments. A total of 54 samples were collected, generating 455.27 Gb of clean data after removing low-quality reads. More than 92.06% of the clean data achieved a Q30 value. The mapping rate to the reference tea plant genome ranged from 84.41% to 86.87% (Table S3). Under the nitrate treatment, the number of differentially expressed genes (DEGs) in ZN117 was higher than that in TGY at all time points except for 12 h. At 72 h, ZN117 had the highest number of DEGs, with 4375 upregulated DEGs and 4192 downregulated DEGs. Under the chlorate treatment, TGY had a higher number of DEGs compared to ZN117 at each time point. Generally, more DEGs were found in the leaves under the chlorate treatment compared to the nitrate treatment, except for the DEGs in ZN117 at 6 h (Fig. 3a). In addition, a total of 3118 TFs were detected in the sequencing results. Among these TFs, 1032, 1413, 2446, and 2023 TFs were identified in nitrate–TGY, nitrate–ZN117, chlorate–TGY, and chlorate–ZN117, respectively, with different expression abundances, compared to the initial time point (0 h). These TFs were classified into 65 families according to the PlantTFDB database. Among them, the bHLH, MYB, and AP2/ERF-ERF families were the most abundant (Fig. S2). A total of 3598 DEGs were common to all four treated groups (Fig. 3b), with 5441 DEGs shared between nitrate–TGY and nitrate–ZN117, and 13 104 shared between chlorate–TGY and chlorate–ZN117. Similarly, 7721 DEGs were shared between nitrate–TGY and chlorate–TGY, and 8649 DEGs were shared between nitrate–ZN117 and chlorate–ZN117. Principal component analysis (PCA) showed that PC1 accounted for 59.9% of the total variance and separated samples based on the nitrate and chlorate treatments, while PC2 accounted for 14.5% of the total variance and separated samples based on different cultivars (Fig. 3c**)**. To validate the RNA-seq data, 26 genes were selected and their expression levels were detected after nitrate and chlorate treatments in both cultivars at 6 and 72 h by quantitative real-time PCR (qRT-PCR). The relative expression fold changes were consistent with the RNA-seq data, confirming the accuracy of the transcriptome analyses according to Pearson correlation tests (*r* = 0.96, *P* < 0.0001) (Fig. S3).

### KEGG enrichment analyses of RNA-seq

KEGG-annotated Kyoto Encyclopedia of Genes and Genomes (KEGG) annotated pathway analysis revealed the differences in the putative functions of DEGs between ZN117 and TGY under different treatments. The significantly enriched pathways (*P* < 0.05) in nitrate and chlorate groups are presented according to their corresponding *P*-values. Under the nitrate treatment, ZN117 had a higher abundance of DEGs involved in nitrogen metabolism (ko00910) at 6 and 12 h, and the enrichment of DEGs involved in nitrogen metabolism was significant in TGY only at 24 and 72 h. In contrast, TGY exhibited major changes in transcriptional activity in pathways related to photosynthesis (ko00195), photosynthesis-antenna proteins (ko00196), and porphyrin and chlorophyll metabolism (ko00860) at different time points. The enrichment factors of these pathways were higher in ZN117. For the chlorate treatment, ZN117 exhibited sensitivity in nitrogen metabolism at 6 h, while the DEGs in TGY were not significantly enriched in this pathway. The enrichment of DEGs was observed in pathways related to porphyrin and chlorophyll metabolism at all time points, except for ZN117 at 12 h. In addition, TGY showed significant enrichment in photosynthesis-antenna proteins at each time point after chlorate treatment, with a higher enrichment factor than ZN117. The photosynthesis pathway showed significantly more enrichment in ZN117 (12, 24, and 72 h) compared to TGY (6, 24, and 72 h) (Fig. S4).

### DEGs involved in chlorophyll metabolism and photosynthesis

**Figure 4:**
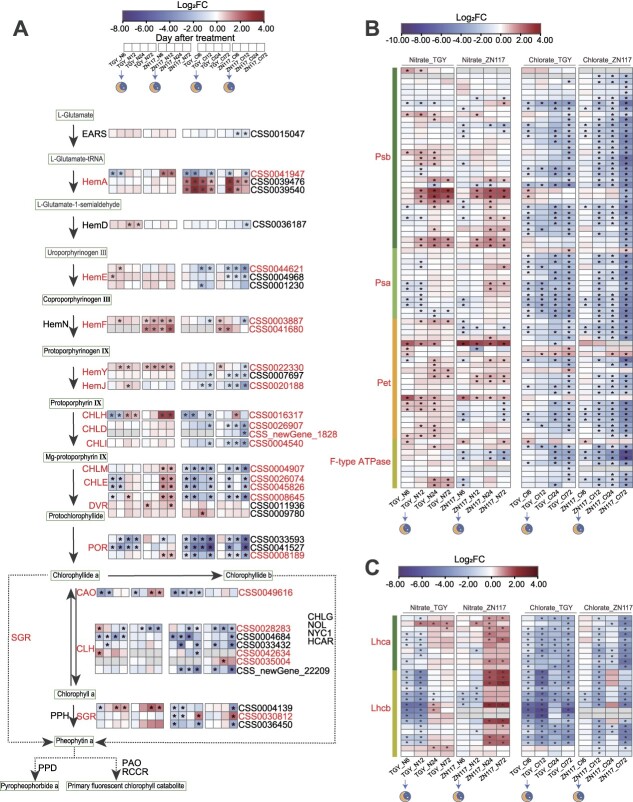
Schematic map of DEGs presented in leaves related to chlorophyll metabolism and photosynthesis. Heat map of DEGs between TGY and ZN117 at different stages after treatments involved in (A) chlorophyll biosynthesis and degradation, (B) photosynthesis, and (C) light-harvesting chlorophyll proteins. N, nitrate treatment; Cl, chlorate treatment. DEGs (fold change >1.5) are presented between TGY and ZN117 at different stages after treatments in the heat map based on log2 fold changes. ^*^ denotes FDR ≤ 0.01.

The symptoms of the chlorate-sensitive assay primarily occur as leaf color changes. Therefore, the DEGs involved in porphyrin and chlorophyll metabolism and photosynthesis were selected for analysis. Table S4 presents a schematic overview of chlorophyll biosynthesis and degradation, with 60 key genes identified to be involved in these pathways. Specifically, genes involved in chlorophyll biosynthesis, including *CsHemA* (*CSS0041947*), *CsPOR* (*CSS0008189*), and *CsCAO* (*CSS0049616*), were upregulated in ZN117 after 24 h of nitrate treatment. However, these genes were downregulated by nitrate in TGY. *CsHemF* (*CSS0003887*, *CSS0041680*), *CsHemY* (*CSS0022330*), *CsCHLH* (*CSS0016317*), and *CsCHLE* (*CSS0026074*, *CSS0045826*) showed a higher level of induction in ZN117 compared to TGY. Moreover, *CsCLH* (*CSS0028283*, *CSS0042634*), which is responsible for chlorophyll degradation, was induced at 6 h in TGY. Genes involved in chlorophyll biosynthesis, including *CsHemE* (*CSS0044621*), *CsHemJ* (*CSS0020188*), *CsCHLD* (*CSS0026907*, *CSS_newGene_1828*), *CsCHLI* (*CSS0004540*), *CsCHLM* (*CSS0004907*), and *CsDVR* (*CSS0008645*), were more strongly downregulated in ZN117 compared to TGY at 72 h under chlorate treatment. *CsSGR* (*CSS0030812*) and *CsCLH* (*CSS0035004*), which are involved in chlorophyll degradation, were increasingly expressed in ZN117 (Fig. 4a). Similarly, the expression levels of light-harvesting chlorophyll genes exhibited a marked increase in ZN117, with NO_3_^−^ as the sole N source. Under chlorate treatment, the transcripts of these genes were downregulated, particularly in ZN117 (Fig. 4b, Table S5). Furthermore, genes involved in photosynthesis were upregulated significantly in ZN117 when exposed to NO_3_^−^ after 24 h. However, the expression of these genes in ZN117 showed a sharp decrease after 72 h of chlorate treatment (Fig. 4c, Table S5). These DEGs may contribute to the observed phenotypic changes in leaf color in response to ClO_3_^−^ in the two cultivars.

### Differentially expressed NRT gene analyses

**Figure 5:**
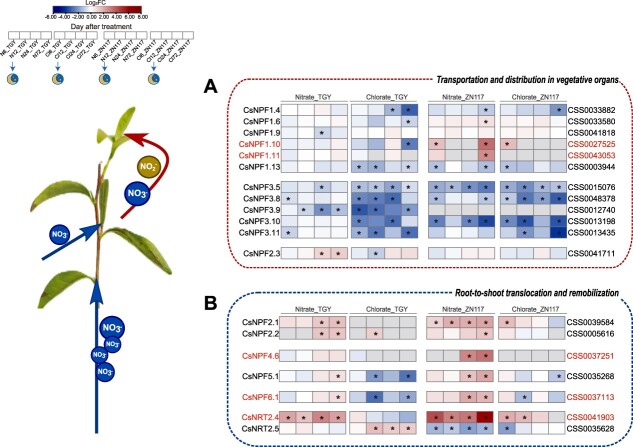
Overview map of nitrate transporter DEGs in leaves of TGY and ZN117 after the nitrate and chlorate treatments. N, nitrate treatment; Cl, chlorate treatment. DEGs (fold change >1.5) between TGY and ZN117 are presented in the heat map at different stages after treatments based on log_2_ fold changes. Gray indicates the absence of DEGs at this time point. ^*^ denotes FDR ≤ 0.01.

To explore the potential regulation of the transport of NO_3_^−^ and ClO_3_^−^ in tea plant branches, Fig. 5 presents the heat maps of the NRT genes. In the process of NO_3_^−^ allocation to young leaves, the expression of *CsNPF1.10* and *CsNPF1.11* were upregulated in ZN117 after the nitrate treatment, while no significant changes were observed in TGY. *CsNPF4.6* and *CsNPF6.1*, responsible for NO_3_^−^ lateral allocation and root–shoot transport, were induced in ZN117 after nitrate treatment and continued to increase over time. These findings provide a molecular basis for the difference in NiUE between the two cultivars. In addition, *CsNPF2.1* and *CsNPF2.2* were both found to be upregulated in TGY and ZN117. The expression of *CsNPF5.1* increased in ZN117 and TGY after 24 and 72 h of NO_3_^−^ application, respectively. In the chlorate treatment, the expression of most *NPF* genes showed a downward trend in both cultivars. Furthermore, the expression of *CsNRT2.4*, which is involved in NO_3_^−^-regulated root and shoot growth, was induced in both TGY and ZN117 after nitrate treatment, with the latter exhibiting a stronger increase (Fig. 5). *CsNRT3.1* showed increased expression in TGY, especially under the chlorate treatment, while no changes were observed in ZN117. The study also investigated the expression of *CLC* genes, which are responsible for encoding chloride channels. *CsCLC-c*, *CsCLC-e*, and *CsCLC-f* showed decreased expression levels in both cultivars following nitrate treatment. In the chlorate treatment, the expression of *CsCLC-e* was downregulated more in ZN117 compared to TGY, while *CsCLC-f* expression increased markedly in TGY. The expression of *CsSLAC1* (slow anion channel-associated 1) showed a noticeable downward trend in the first 12 h after both treatments, while *CsSLAH1* (SLAC1 homolog 1) increased strongly in both cultivars after 12 h of chlorate treatment (Fig. S5b). Furthermore, the transcripts of *NRT* genes—involved in NO_3_^−^ distribution in reproductive organs and stress responses—were also regulated by nitrate treatment in both cultivars (Fig. S5, Table S6).

### DEG analyses in nitrogen metabolism

**Figure 6:**
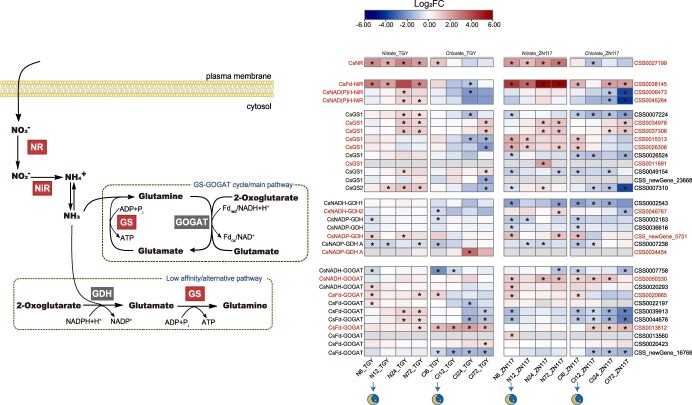
Overview map of nitrogen metabolism DEGs in leaves of TGY and ZN117 after the nitrate and chlorate treatments. N, nitrate treatment; Cl, chlorate treatment. DEGs (fold change >1.5) are presented in the heat map between TGY and ZN117 at different stages after treatments based on log_2_ fold changes.Gray indicates the absence of DEGs at this time point. ^*^ denotes FDR ≤ 0.01.

To understand the differences between ZN117 and TGY in NO_3_^−^ metabolic reduction and ammonia assimilation, we investigated the transcriptional profiles of genes involved in nitrogen metabolism (Fig. 6, Table S7). The results showed that ZN117 had a stronger upregulation (fold change = 8.63, log_2_FC = 3.11) of *CsNR* expression compared to TGY after 72 h of nitrate treatment. In addition, the expression of *CsFd-NiR*, which is involved in NO_3_^−^ reduction, was also upregulated more in ZN117 compared to TGY at 6 h (fold change = 22.3, log_2_FC = 4.48) and 24 h (fold change = 51.6, log_2_FC = 5.69). The changing trends were generally consistent in both the nitrate-treated and chlorate-treated groups in ZN117. In TGY, *CsFd-NiR* showed a downregulation after 24 h of chlorate treatment. In contrast, *CsNAD(P)H-NiR* expression increased after 24 h of nitrate supply in TGY, and decreased significantly after 24 h of chlorate treatment in both cultivars. Furthermore, compared to TGY, ZN117 exhibited a higher upregulation of *CsGS* (*CSS0034978*, *CSS0037306*, *CSS0015313*, *CSS0026308*, and *CSS0011691*), a gene encoding the rate-limiting enzyme GS in nitrogen assimilation, during both treatments. The transcription of *CsGDH* (*CSS0046767* and *CSS_newGene_5751*) was also induced to a higher level in ZN117 after nitrate supply. When treated with chlorate, *CsNADP-GDH* (*CSS0034454*) showed the strongest induced effect in TGY at 24 h. *CsNADH-GOGAT* (*CSS0050330*) was highly upregulated in ZN117 under both treatments. The genes of *CsFd-GOGAT* expression were highly promoted in TGY after nitrate treatment. For the chlorate treatment, two genes (*CSS0020665* and *CSS0013812*) were highly induced in both cultivars.

### WGCNA analysis identified key TFs related to N metabolism between two cultivars

**Figure 7:**
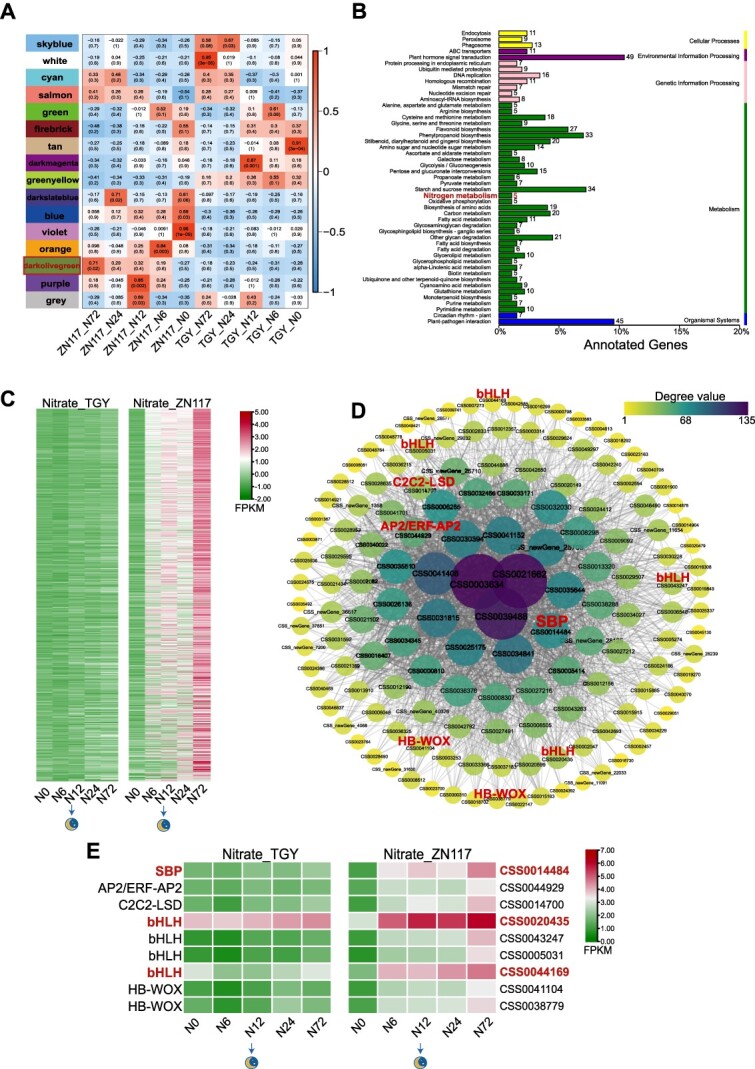
WGCNA based on the DEGs in TGY and ZN117 leaves after treatment with nitrate. (A) Module–trait correlation analysis. Each column corresponds to a sample group. Pearson’s coefficient between the module and sample is described by each block at the row–column intersection. (B) KEGG enrichment of DEGs. Numbers beside the bar indicate the number of DEGs in this pathway. (C) Heat maps of the expression profiles of coexpressed genes in the ‘darkolivegreen’ module. (D) Gene coexpression network for the ‘darkolivegreen’ module. The network presents 146 genes in this module, selected based on connectivity in the top 18% and edge weights ≥0.35. Node size and color are proportional to the number of edges and degrees of coexpressed genes. (E) Heat maps of the expression profiles of nine selected TFs after the nitrate treatment. N, nitrate treatment; Cl, chlorate treatment.

To obtain a comprehensive understanding of the specific genes related to N metabolism between TGY and ZN117, we performed a weighted gene coexpression network analysis (WGCNA) based on the DEGs when NO_3_^−^ supply in both cultivars and 16 modules were identified and labeled with different colors. Among these modules, the ‘darkolivegreen’ module in ZN117 exhibited a strong correlation with the response to nitrate treatment and was gradually upregulated (Fig. 7a). This module contained 1119 DEGs and KEGG enrichment showed that the coexpressed genes, including *CsNRT2.4* (*CSS0041903*), *CsGDH* (*CSS0046767*), and *CsGS* (*CSS0011691*), were significantly enriched in the nitrogen metabolism pathway (Fig. 7b, c). To identify the TFs regulating N metabolism, a network was constructed using the edge weights ≥0.35 and included 2079 relationships (Fig. 7d). The size and color of the nodes in the network represented the number of edges and degrees of each gene. Among the identified TFs, nine were annotated to five families, including *SBP* (*CSS0014484*, 71 edges), *AP2/ERF-AP2* (*CSS0044929*, 51 edges), *C2C2-LSD* (*CSS0014700*, 34 edges), *bHLH* (*CSS0020435*, 28 edges; *CSS0043247*, 27 edges; *CSS0005031*, 24 edges; *CSS0044169*, 9 edges), and *HB-WOX* (*CSS0041104*, 19 edges; *CSS0038779*, 10 edges). After nitrate treatment, the transcripts of *SBP* (*CSS0014484*) and *bHLH* (*CSS0020435* and *CSS0044169*) were significantly increased in ZN117 but remained relatively unchanged in TGY (Fig. 7e Table S8). Interestingly, under chlorate treatment, these three genes were also highly upregulated in ZN117. The background fragments per kilobase of transcript per million fragments mapped (FPKM) of *CSS0020435* was high and its expression was not influenced by NO_3_^−^, yet it was reduced by ClO_3_^−^ at 12 and 72 h (Fig. S6c).

## Discussion

### Fast identification of nitrate use differences in tea plants using leaf chlorate sensitivity

NO_3_^−^, which serves as the main form of inorganic N in soils, is typically lost through leaching in tea gardens [20, 32]. Thus, enhancing NiUE is a critical step toward achieving sustainable tea production. ClO_3_^−^ is a herbicide, defoliant, and a toxic analog to NO_3_^−^ [33]. The use of chlorate as a method for assessing NiUE in crops has been proven to be effective, especially in cereal plants with shorter growth cycles, such as barley [15], rice [16, 1,7], and wheat [18]. These plants may show signs of toxicity, such as alterations in plant height or leaf morphology, following treatment with ClO_3_^−^. For example, in rice, plant height was used to determine growth status, and the percent inhibition of growth was calculated as the ClO_3_^−^ sensitivity [16, 17, 34, 35]. In barely, leaf chlorosis under KClO_3_ treatment was visually scored to assess ClO_3_^−^ resistance [15]. In this study, we attempted to apply the ClO_3_^−^-sensitive assay to woody perennial plants to evaluate NiUE. To study NO_3_^−^ distribution in new leaves, the branches were grown in a solution in which KNO_3_ was replaced by KClO_3_. Under this condition, nutrient uptake relied on transpiration pull. Thus, two cultivars—ZN117 (high sensitivity) and TGY (low sensitivity)—with similar transpiration rates were selected from a total of 10 cultivars (Fig. 2a). The results showed that the values of SPAD (foliar chlorophyll concentration [29]) and F_v_/F_m_ (sensitivity of photosystem II to environmental stress [30]) in ZN117 decreased continuously after 2 days of ClO_3_^−^ treatment, reaching significant levels (Fig. 1and Fig. S1). This indicates that ZN117 has lower ClO_3_^−^ resistance compared to TGY. Furthermore, ^15^N labeling results confirmed that ZN117 had a stronger capability to transport ^15^NO_3_^−^ to young shoots than TGY (Fig. 2b–d). More importantly, the measurements of SPAD and F_v_/F_m_ provide rapid and noninvasive assessments of leaf chlorophyll levels and sensitivity to environmental stress, which allows for the real-time monitoring of plant toxicity. The expression of genes involved in chlorophyll biosynthesis exhibited a strong decrease in ZN117, contributing to the yellowing phenotype observed in leaves after the chlorate treatment [36]. These results demonstrate the strong applicability of chlorate resistance for the rapid assessment of tea plants and its potential role as a reference for other woody plants.

**Figure 8:**
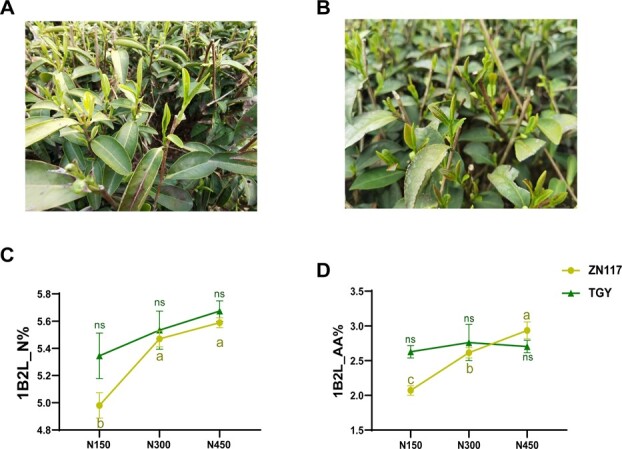
Phenotypic observations, nitrogen content, and total free AA contents of 1B2L in TGY and ZN117 under different nitrogen fertilizer applications. Phenotypic characterization of 1B2L in (A) ZN117 and (B) TGY. (C) Total nitrogen and (D) free AA contents in 1B2L in both cultivars at different nitrogen levels. Both cultivars were planted with N fertilizer (urea) rates of 150 kg ha^−1^ (N150), 300 kg ha^−1^ (N300), and 450 kg ha^−1^ (N450) in four applications per year. Bars represent the mean ± SE (*n* = 3) with *t*-tests at *P < 0.05 and **P < 0.01.

To further validate the feasibility of the chlorate sensitivity assay using tea branches, 11 varieties with different NiUE levels were selected based on their daily F_v_/F_m_ variations after KClO_3_ treatment. In the sensitive group (A, B, C, D, E, and F), yellowing young leaves, brown leaf veins, and withered leaf margins were observed (Fig. S7a), while in the tolerant group, young leaves turned slightly yellow, and most new shoots continued to sprout (Fig. S7b). There was a negative correlation between the FPKM of *NPF2.2* and *bHLH* and F_v_/F_m_ variation, and a positive correlation between the FPKM of *Fd-NiR* and F_v_/F_m_ variations (Fig. S7c–e). Furthermore, RNA-seq data from the third leaves of these 11 varieties under normal conditions revealed that the FPKM of *NPF2.2* was significantly higher in the sensitive group compared to the tolerant group (Fig. S7f). Field experiments showed that the total N and total free AAs increased significantly with increasing N fertilizer application in young shoots of ZN117, while no differences were observed in TGY (Fig. 8). The highly responsive result in ZN117 suggests that N transfer to developmental tissue in ZN117 was more active, and TGY was found to be more tolerant to barrenness and poorly mobilized to N fertilizer. This is consistent with the results from the ^15^N isotope tracing experiment (Fig. 2). These results further indicate that chlorate resistance can be used as a tool for assessing the NiUE in tea plants. Genes significantly correlated to F_v_/F_m_ variation were proposed here as marker genes for NiUE, and their functions *in planta* require further investigation.

**Figure 9:**
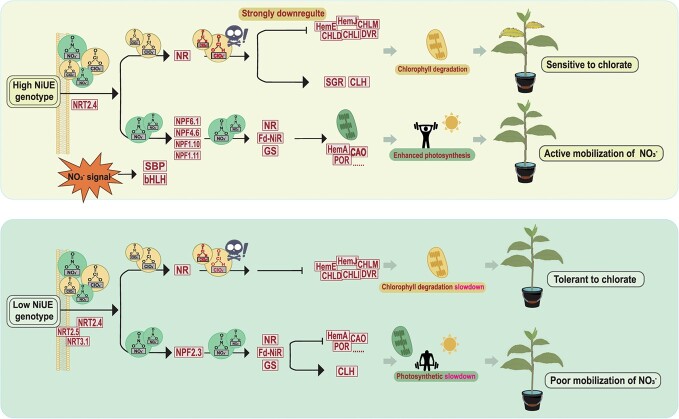
A proposed model for transport and remobilization of nitrate and chlorate in tea plant.

### Genes involved in NO_3_^−^ transport and assimilation exhibited transcriptional differences between two cultivars

NRTs, located in the transmembrane, are mainly involved in NO_3_^−^ uptake and transfer between tissues and cells. NO_3_^−^ has to be reduced into NO_2_^−^ and converted into NH_4_^+^ by NR and NiR to be further incorporated into AAs. In this process, both excess NO_2_^−^ and NH_4_^+^ are toxic to plant cells, and NO_3_^−^ can be stored in vacuoles and retrieved when needed [37]. Therefore, different NiUE cultivars can respond differently to the external NO_3_^−^ supply to determine how much NO_3_^−^ will be stored, transported, or remobilized for normal plant growth, and ultimately transmit information to activate the corresponding transporters or enzymes through transcription regulation [38]. In addition to serving as an N source, NO_3_^−^ can also act as a signal to regulate the expression of the genes required for its use by the plant [39]. NO_3_^−^ has been shown to induce the transcriptional response of *NRT*, *NR*, and *NiR*. This transcriptional regulation can rapidly accumulate mRNA and is regarded as the primary nitrate response (PNR) [38, 40, 41]. In addition, ClO_3_^−^ can be taken up via the same transporter for NO_3_^−^ and serves as a substrate for NR. However, it is not able to mimic NO_3_^−^ as a signal to trigger the transcripts of downstream transporter and enzyme genes [42]. Our results indicate that the changes in the responses of genes such as *NRTs*, *CLCs*, *NR*, and *NiR* after chlorate treatment in both TGY and ZN117 did not align exactly with those after nitrate treatment (Figs. 5 and 6). In the following, we discuss the varying responses to NO_3_^−^ application between ZN117 and TGY to explore the potential mechanism regulating NO_3_^−^ transportation, allocation, and reduction

1. ***NPF subfamily***

The NPF subfamily encompasses 109 nitrate and peptide transporters in the tea plant genome. The expressions of its members show different responses to NO_3_^−^ supply [4]. AtNPF6.3 (AtNRT1.1/AtCHL1) is a bidirectional transporter involved in xylem loading and root stele unloading and plays a crucial role in the root-to-shoot transfer of NO_3_^−^ [43,44]. *CsNPF6.1*, the homology of *AtNPF6.3*, showed increasing transcriptional levels in ZN117 after 24 and 72 h of nitrate treatment (Fig. 5). This is consistent with previous research on ZhongCha302 [20]. The expression of *NPF6.3* induced by NO_3_^−^ has also been reported in *Arabidopsis*, rice, and maize [16, 33, 45]. In addition, the transcripts of *CsNPF4.6*, a homolog of *AtNPF4.4* involved in monitoring internal NO_3_^−^ allocation to regulate shoot architecture at low NO_3_^−^ levels [46], also exhibited high expression induced by nitrate in ZN117. Both the ^15^N distribution and induced expression of *NPFs* indicate that ZN117 was more active than TGY in mobilizing NO_3_^−^ for xylem loading and distributing NO_3_^−^ for new shoot development. Previous studies revealed the induction of the homology gene of *AtNPF2.13* in tea plants, *CsNPF2.3* (*CsNRT1.7*), after a 72-h resupply of nitrate, indicating its analogous role in the remobilization of stored NO_3_^−^ from old leaves to young shoots by phloem loading at low NO_3_^−^ levels as *AtNPF2.13* [20, 47]. In our study, *CsNPF2.3* was upregulated after 24 h of nitrate treatment in TGY. This suggests that the treated NO_3_^−^ concentration was limited for this variety and it required the further mobilization of the stored NO_3_^−^ to support the demand of young shoots. When NO_3_^−^ supply is ample, the xylem-to-phloem transfer of NO_3_^−^ from older leaves to developing tissues is meditated by AtNPF1.1(AtNRT1.12) and AtNPF1.2 (AtNRT1.11) [48]. In *Lycopersicum esculentum*, *NPF1.2* was reported to be highly upregulated in high-NUE genotypes [49]. Similarly, in our study, *CsNPF1.10* and *CsNPF1.11*, the homologies of *AtNPF1.2*, exhibited increased expression by 13.5- and 5.35-fold in ZN117, respectively, suggesting their role in remobilizing NO_3_^−^ from older tissues to feed developing leaves in high-NiUE genotypes. When treated in chlorate, *CsNPF* expression almost always significantly decreased, implying that ClO_3_^−^ may not transfer to younger tissues. However, *CsNPF1.10* was upregulated in ZN117 after short-term chlorate supply, indicating that ClO_3_^−^ may accumulate more in ZN117 than TGY and have a tendency to transfer to new shoots.

1. ***NRT2, CLC*, *and SLAC/SLAH subfamilies***

NRT2 transporters are known to only transport NO_3_^−^ and play a major role in NO_3_^−^ acquisition and root-to-shoot translocation [11, 50–52]. The expression of *CsNRT2.4* and *CsNRT2.5* regulated by chlorate treatment indicated that NRT2 may transport ClO_3_^−^ due to the similar structure of ClO_3_^−^ with NO_3_^−^. Scholars have reported *CsNRT2.4* to be mainly expressed in roots, playing a major role in NO_3_^−^ uptake [20, 53]. In *Arabidopsis*, AtNRT2.4 influences the NO_3_^−^ content in shoot phloem exudates, and AtNRT2.5 assists AtNRT2.1, AtNRT2.2, and AtNRT2.4 in the phloem loading of NO_3_^−^ [54,55]. In tea plants, the expression of *CsNRT2.4* increased dramatically in the high-NiUE genotype, while *CsNRT2.5* showed a decreasing trend. Previous research showed that *CsNRT3.1* (*CSS0034664*) had a relatively high expression level in leaves [16], and we found it to be induced by a short-term resupply of NO_3_^−^. In *Arabidopsis*, the coexpression of *AtNRT3.1* is required for the initiation of the high-affinity transport system meditated by NRT2 [56], and AtNRT2.5 can collaborate with other NRT2s to ensure efficient NO_3_^−^ transport in N-starved plants [55]. In our study, *CsNRT3.1* and *CsNRT2.5* were highly induced to mobilize NO_3_^−^ for its subsequent redistribution *in vivo* after chlorate treatment. This was also found under N-deficiency conditions (Fig. 5 and Fig. S5a).

1. ***Enzymes for NO***_***3***_^***−***^ ***reduction and assimilation***

During NO_3_^−^ reduction, the expression of *NiR* is strongly induced by NO_3_^−^ to avoid NO_2_^−^ toxicity [57]. Our study revealed that the increasing *NiR* transcript was even greater than that of *NR*, especially in ZN117. Moreover, *CsFd-NiR* expression was steeply upregulated after both nitrate and chlorate treatments (Fig. 6), suggesting that this chlorate-sensitive cultivar may produce higher concentration substrates of NO_2_^−^ and ClO_3_^−^, leading to a higher reduction ability. In addition to NO_3_^−^, the transcriptional regulation of *NR* and *NiR* is also induced by light, exhibiting diurnal variation [58, 59]. There was a small decrease in the expression levels of both cultivars after 12 h of NO_3_^−^ treatment at night. This upward trend in expression was also evident in the gene *CsGS*, the fundamental role in N assimilation [60], suggesting that ZN117 may possess a greater ability to assimilate AAs compared to TGY.

### Transcriptional activation by NO_3_^−^ signal transduction may be regulated by SBP and bHLH

Squamosa promoter bind protein-like 9 (SPL9) is predicted to be a potential hub affecting expression levels of genes including *NR* and *NiR* [61]. Here, an identified WGCNA network revealed that one of the *CsSBP* was induced dramatically by the NO_3_^−^ signal, while two *CsbHLH* genes showed strong induction after NO_3_^−^ supply (Fig. 7e). This may be because *CsbHLH* (*CSS0020435*), with the highest homology of *bHLH134*/*135*/*136* in *Arabidopsis*, plays major roles in response to light and phytohormones pathways, and activating its transcription affects cell elongation, flowering time, and chlorophyll levels in plants [62–65]. In our study, *CSS0020435* revealed a relatively higher FPKM value and was markedly induced, especially in ZN117. Numerous studies have revealed that some NPFs transport both NO_3_^−^ and plant hormones [66–68]. Therefore, we speculate that multiple phytohormone levels may change during NO_3_^−^ acquisition, transfer, and remobilization, thus activating the downstream *CsbHLH* transcription. These results provide the molecular basis for the application of the chlorate-sensitive assay to identify differences in NO_3_^−^ mobilization. However, further research on the gene functions is required to fully understand the mechanisms underlying these processes and their implications for plant growth and development.

## Conclusion

This study conducted a chlorate-sensitive assay in tea plants using short-time branch hydroponics among different cultivars. The high- and low-sensitivity cultivars, ZN117 and TGY, respectively, were selected based on the temporal responses of SPAD and F_v_/F_m_. Isotope labeling results for ^15^NO_3_^−^ confirmed the presence of a difference in the NO_3_^−^ distribution between two cultivars. Based on transcriptomic analysis, we proposed a speculative model for NO_3_^−^ transfer in the high-NiUE genotype. NO_3_^−^ supply may alter phytohormone levels, thus activating downstream *bHLH* TF-mediated signaling pathways. Simultaneously, SBP in response to NO_3_^−^ signals affects the NO_3_^−^-driven gene network. *CsNRT2.4* and *CsNPF6.1* are involved in transferring NO_3_^−^ to mature leaves through xylem loading, while *CsNPF4.6* acts as a potential transceptor to monitor and allocate internal NO_3_^−^ near the xylem. *CsNPF1.10* and *CsNPF1.11*, on the other hand, redistribute NO_3_^−^ to young shoots via the xylem-to-phloem transfer route. Furthermore, the low-NiUE genotype further remobilized stored NO_3_^−^ by *CsNPF2.3* for subsequent utilization. NO_3_^−^ in vigorous tissues can be further reduced and assimilated by key enzyme genes related to anabolism (*NR*, *NiR*, and *GS*). At the same time, genes responsible for chlorophyll synthesis are upregulated, thus enhancing photosynthesis and providing energy for plant growth. When chlorate is supplied, the analog is mainly transported by *NRT2.4*. *NRT2.5* and *NRT3.1* are also induced to assist in the involvement of transporter particular for the low-NiUE variety. This process accelerates chlorophyll degradation, resulting in a yellowing and withering phenotype and a damaged photosynthetic system. These key genes in NO_3_^−^ transport and metabolism may be potential marker genes for identifying efficient nitrate utilization. In the future, additional cultivars can be tested to correlate the transcription of such genes with chlorate toxicity phenotypes for the screening and validation of more indicative and widely adaptive genes related to NO_3_^−^ utilization (Fig. 9).

## Materials and methods

### Plant materials and treatments

Branches of 10 tea cultivars were collected in August 2021 from 8-year-old tea plants in the Experimental Station of the Tea Research Institute of the Chinese Academy of Agricultural Sciences located at Shengzhou, Zhejiang Province (29.74°N, 120.82°E). The 10 cultivars were Baiye 1 (BY1), Dangui (DG), Fuding Dabaicha (FD), Huangjinya (HJY), LongJing 43 (LJ43), Tieguanyin (TGY), Jiaming 1 (JM1), Zhongcha 108 (ZC108), Zijuan (ZJ), and Zhenong 117 (ZN117). These cultivars were uniformly managed with the same practices of fertilization, pruning, irrigation, and harvesting since planting. Branches grown from spring, with a 40- to 45-cm length, six to seven mature leaves, and two sprouting top leaves were collected for testing. Approximately half of the stem was lignified, while the remaining half remained green. The ends of the branches were immediately placed into deionized water after cutting and were then transported to the laboratory, where they were planted in black pots filled with 5 l of nutrient solution in a growth chamber. Each pot contained six branches and was continuously ventilated. The half-strength nutrient solution contained the following macronutrients (mM): P, 0.05; K, 1.05; Mg, 0.2; Ca, 0.4; S, 0.2 and micronutrients (μM): Fe, 3.15; B, 5; Mn, 0.75; Zn, 0.5; Cu, 0.1 and Mo, 0.25 [69]. Two treatments were performed: the control group contained 1 mM KNO_3_; and the chlorate group contained 1 mM KClO_3_ as a replacement for the KNO_3_. Each treatment was replicated three times. The pH of the nutrient solution was adjusted daily to 5.0 ± 0.5 using NaOH and H_2_SO_4_. The growth chamber had day/night temperatures of 30°C/25°C and a light/dark cycle of 14/10 h. After selecting the two contrasting cultivars (TGY and ZN117) based on their chlorate resistance, a new round of branch hydroponic cultivation was performed repeatedly in both cultivars. The cultivation conditions were set up as before, except that K^15^NO_3_ (at 10% atom excess) was replaced with KNO_3_.

Samples of one bud with two leaves (1B2L) were collected on 27 March from ZN117 and 12 April from TGY (spring tea harvest season of 2024) growing in a long-term N addition field experiment at the Shengzhou Experimental Station. Both cultivars were planted in 2015, with N fertilizer (urea) rates of 150 kg ha^−1^ (N150), 300 kg ha^−1^ (N300), and 450 kg ha^−1^ (N450) applied four times per year: early February (30% of total), May (30%), June (20%), and October (20%). In the verification experiment, 11 varieties of 3-year-old seedlings were grouped based on their disparity in chlorate resistance. The varieties were denoted A–K and divided into sensitive (A, B, C, D, E, and F) and tolerance (G, H, I, J, and K) groups.

### Sampling and measurements

The relative chlorophyll contents of the third youngest fully expanded leaves of 10 cultivars were measured at 1, 2, 3, 4, and 5 days after cultivation in treated nutrient solution using a SPAD-502 Plus chlorophyll meter (Spectrum Technologies, Konica Minolta, Japan) [29]. Each leaf was measured at six sites (excluding the main vein) and the values were averaged. The chlorophyll fluorescence of the third leaves was noninvasively measured at the aforementioned time points using a FluorPen FP110 portable fluorometer (Photon Systems Instruments, Czech Republic).

The pots containing nutrient solutions were weighed at the start and end (5 days) of the experiment. The transpiration rate (TR) was calculated according to equation (1) [70]:


(1) \begin{equation*} \mathrm{TR}={\mathrm{FW}}_{\mathrm{b}}/\left( Wt1- Wt2\right), \end{equation*}

where FW is the fresh weight of the branches and *Wt*1 and *Wt*2 are the weights of the nutrient solution at the start and end of the experiment, respectively.

In the cultivated experiment of ZN117 and TGY with K^15^NO_3_, the third leaves from the top of the branches were sampled at 0, 6, 12, 24, 72, and 120 h. All samples were stored at −80°C for further analysis. At the end of the experiment (120 h), other leaves and the stem were separately collected and divided into two parts, one including the lignified stem and old leaves and the other including the green stem and leaves. The stem and leaves were dried in a vacuum stoppering tray dryer (Labconco, USA) until a constant weight was reached. The samples were then ground into a fine powder for the determination of ^15^N abundance using a MAT253 isotope ratio mass spectrometer (Thermo Finnigan, Germany) coupled to an elemental analyzer (Carlo Erba, Milano, Italy). Nitrogen derived from the nutrient solution (N_dff_%) was calculated according to equation (2):


(2) \begin{equation*} \mathrm{Ndff}\%=\frac{{{}^{15}N}_{sample}-{{}^{15}\mathrm{N}}_{\mathrm{natural}}}{{{}^{15}N}_{solution}-{{}^{15}\mathrm{N}}_{\mathrm{natural}}}\times 100, \end{equation*}

where ^15^N_sample_ is the ^15^N abundance measured in the sample, ^15^N_solution_ is the ^15^N abundance (10%) in the nutrient solution and ^15^N_natural_ is the natural ^15^N abundance.

The accumulation of ^15^N in the leaves, stem, and branches was calculated as follows:


(3) \begin{equation*} {}^{15}N\ accumulation= Ndff\%\times total\ N\ concentration. \end{equation*}

where the total N concentration is the total N concentration measured by the elemental analyzer in the sample.

The allocation ratio of ^15^N in leaves and stems was calculated according to equation (4):


(4) \begin{equation*} {}^{15}N\ ratio\ in\ leaves\ or\ stem=\frac{{}^{15}N\ accumulation\ in\ leaves\ or\ stem}{{}^{15}N\ accumulation\ in\ branch}. \end{equation*}

### Determination of total N and free amino acids

The total N concentration of 1B2L was determined using an elemental analyzer (Vario Max CN Analyzer, Elementar Analysensysteme GmbH, Germany) by measuring the N_2_ released after combustion at 950°C. A total of 0.1 g dried sample was extracted in 5 ml deionized water for 5 min in a 100°C bath to prepare the extracting solution. The total free AAs were measured based on the ninhydrin method with glutamic acid as a standard [71].

### RNA isolation, complementary DNA (cDNA) library construction, and Illumina RNA-sequencing

Leaf samples were collected from ZN117 and TGY treated with nitrate and chlorate at 0, 6, 12, 24, and 72 h with three replicates. Total RNAs were extracted using the RNAprep Pure Plant Kit (DP411, Tiangen, China) following the manufacturer’s protocol. The RNA integrity was assessed using the Agilent 2100 LabChip GX (Perkinelmer, USA) and the RNA concentration was measured using Nanodrop 2000 (Thermo Fisher Scientific, USA). A total of 2 μg of RNA per sample was used to construct the cDNA library using a VAHTS Universal V6 RNA-seq Library Prep Kit for Illumina (NR604–0, Vazyme, China), and the cDNA library was purified using VAHTSTM DNA Clean Beads (N411–03, Vazyme, China). The libraries were sequenced on an Illumina NovaSeq 6000 platform.

### RNA-seq data analysis

Raw reads containing adapter, poly-N, and low-quality reads were removed to obtain clean reads. All sequence clean reads were mapped to the reference genome tea plant for Shuchazao (*Camellia sinensis var. sinensis*) [72] using Tophat2 [73]. FPKM were used to estimate gene expression levels in StringTie (v2.2.3) [74]. The average gene expression levels of three replicated at each time point (6, 12, 24, and 72 h) for TGY and ZN117 were compared with the CK (0 h) under two treatments, respectively. DEGs were determined by applying DEseq2 with the log_2_ fold change (log_2_FC) ≥ 1.5 and false discovery rate (FDR) ≤ 0.01. KEGG ortholog (KO) database enrichment analysis of the DEGs was performed using the clusterProfiler R package (v1.6.0) (R Core Team) and KO-based annotation system (KOBAS) [75]. Coexpression networks were constructed using WGCNA (v1.66) package in R [76]. The networks were visualized using Cytoscape v3.7.2 (https://cytoscape.org/).

### Validation of RNA-seq data by quantitative real-time PCR

Based on the transcriptome results, a total of 26 DEGs expressed in TGY and ZN117 with NO_3_^−^ and ClO_3_^−^ treatments after 6 and 72 h were validated using qRT-PCR. Primers were designed using Primer Premier 5 and DNAman crossed intron region. The primer specificity was tested by the BLAST program from NCBI (http://www.ncbi.nlm.nih.gov/tools/primer-blast/) and TPIA (http://tpia.teaplant.org), and validated by calculating the primer efficiency between 95% and 105%. *CsGAPDH1* (KA295375.1) was chosen as a housekeeping gene [77]. The primer information is reported in Table S9. The PrimeScript™ RT reagent Kit with gDNA Eraser (Takara, Japan) and SYBR Green™ Premix Ex Taq™ II (Takara, Japan) were used to conduct the reverse transcription and qPCR reactions. Fluorescence data was collected using the LightCycler 480 Instrument II (Roche, USA). Triplicate biological and technical experiments were performed for each sample, and the relative expression levels were calculated using the 2^−ΔΔCt^ method.

### Statistical analysis

To analyze the data obtained from the experiments, statistical analyses were performed using SPSS Statistics 26.0 (IBM Corp.). One-way analysis of variance (ANOVA) was used to compare the means of multiple groups, and the means of two groups were compared using independent *t*-tests. Pearson correlation tests were employed to determine the correlations between F_v_/F_m_ variations and gene FPKM values. The confidence and prediction bands in the linear regression were established at the 95% level.

## Data Availability

RNA-Sequencing data in this study can be found at Sequence Read Archive of NCBI (https://www.ncbi.nlm.nih.gov/sra), the accession number SRA: PRJNA1066199.
